# Effect of octanoic acid-rich formula on plasma ghrelin levels in cachectic patients with chronic respiratory disease

**DOI:** 10.1186/1475-2891-8-25

**Published:** 2009-06-16

**Authors:** Jun-ichi Ashitani, Nobuhiro Matsumoto, Masamitsu Nakazato

**Affiliations:** 1 Third Department of Internal Medicine, Miyazaki University School of Medicine, Kihara 5200, Miyazaki 889-1692, Japan

## Abstract

**Background:**

For cachectic patients with chronic respiratory disease (CRD), conventional enteral nutrition formula is an optional treatment to maintain energy balance. The molecular mechanisms by which enteral nutrition formula controls appetite and weight remain unknown. We examined whether enteral nutrition formula rich in octanoic acids would increase plasma levels of ghrelin, an appetite-stimulating hormone produced in the stomach, in cachectic patients with CRD.

**Methods:**

Plasma ghrelin profiles in cachectic patients with CRD were assessed and compared with those in age- and sex-matched controls. Plasma levels of acyl-ghrelin, an active ghrelin modified by octanoic acids, and desacyl-ghrelin were measured separately. We examined changes in 24-h plasma ghrelin profiles before and after single administration of the formula. We also evaluated the effects of 2-week administration of the formula on plasma ghrelin levels and nutritional status in patients.

**Results:**

The ratio of acyl-ghrelin to desacyl-ghrelin in plasma was lower in patients than in controls. Single administration of the formula did not change plasma desacyl-ghrelin levels, but induced an increase in acyl-ghrelin levels. Two-week treatment with the formula was effective in increasing weight and acyl-ghrelin, along with improving nutritional status in patients.

**Conclusion:**

These results show that the formula contributes to increased weight, which may be associated with induction of acyl-ghrelin production in cachectic patients with CRD.

## Background

Weight loss and nutritional depletion represent independent risk factors for the incidence of pneumonia and mortality in patients with chronic respiratory diseases (CRD) [1,2]. Excess energy expenditure and appetite loss are the main causes of weight loss in such patients, and are difficult to control using established treatments. Enteral nutrition formula is often used as a supplement for patients with insufficient oral calorific intake, although the effects of additional nutrition on weight gain seem to differ depending on the components of supplementation [3,4]. The contribution of formula components to weight gain and to induction of orexigenic hormones remains unclear.

Ghrelin, a novel growth hormone (GH)-releasing peptide, was first isolated from the stomach [5] and induces a positive energy balance by stimulating food intake through GH-independent mechanisms. Acyl-ghrelin, an active ghrelin that induces appetite through the hypothalamus, is synthesized in the stomach and inactivated as desacyl-ghrelin by deacylation. Octanoic acids are essential for acylation in the biosynthesis of acyl-ghrelin. Increased intake of octanoic acids may thus increase plasma acyl-ghrelin levels. Many reports have provided molecular analysis of ghrelin in patients with malignancy, but few have analyzed ghrelin levels in cachectic patients with CRD.

Based on the hypothesis that octanoic acids are necessary for acylation in biosynthesis of acyl-ghrelin, we investigated whether oral administration of an octanoic acid-rich formula would increase plasma acyl-ghrelin levels in cachectic patients with CRD.

## Methods

### Participants

We recruited 4 inpatients (2 women, 2 men; age range, 62–72 y; 2 smokers, 2 ex-smokers; duration of the illness, 2–5 y; BMI, 15.8 ± 1.7) and 19 outpatients (8 women, 11 men; age range, 62–78 y; 7 smokers, 12 ex-smokers; duration of illness, 1–10 y; BMI, 16.0 ± 2.0) with CRD. Underlying pathology was bronchiectasis in 2 and 7 patients, COPD in 1 and 7 patients, and old pulmonary tuberculosis in 1 and 5 patients, respectively. At enrolment, the following inclusion criteria were applied: i) stable respiratory disease for >6 months; and ii) cachexia with complaints of appetite loss. The following exclusion criteria were adopted: i) treatment with steroids, immunosuppressants or antibiotics prescribed within 3 months prior to the study; or ii) presence of pneumonia, cancer or asthma. Cachectic patients were defined as those with documented nonedematous and nonintentional weight loss >7.5% of previous normal weight over a period of ≤ 6 months and body mass index (BMI) <21 at entry. All patients provided written informed consent for participation and the Research Ethics Committee of Miyazaki University approved all study protocols in advance.

### Study protocol

The present study set 2 protocols, as described below. First, we investigated the difference in 24-h profiles for plasma ghrelin levels with and without administration of an enteral nutrition formula rich in octanoic acids using 4 inpatients with CRD on admission. The enteral nutrition formula used here provides 3.0 g of octanoic acid triglyceride and 400 kcal per 400 ml (EN Otsuka, Naruto, Japan). The formula was prepared to provide 2.8 g/day of octanoic acid to patients when tricaprylin hydrolyzed by lipase and free octanoic acid become 100% detached. On day 1, blood samples were taken from the 4 inpatients with calorie intake limited to 1,800 kcal/day. On day 2, 400 ml of the formula was administered between breakfast and lunch in addition to meals providing 1,800 kcal. Blood samples were drawn at 07:00, 09:00, 12:00, 14:00, 17:00, 19:00 and 21:00 to identify 24-h profiles of plasma ghrelin levels. As a second trial, 400 ml/day of formula was orally administered to 19 outpatients for 2 weeks. Body weights of patients were measured at baseline and after 2 weeks of formula administration. Blood samples for these patients were taken on an empty stomach before breakfast to evaluate nutrition status and plasma ghrelin levels at baseline and after 2 weeks of formula administration. Ten age- and sex-matched healthy volunteers were recruited as controls to compare ghrelin levels with those in cachectic patients at baseline. Mean BMI was significantly higher in controls (20.4 ± 5.7) than in patients (p < 0.05).

### Blood sampling and assay

Blood samplings were performed at baseline and during the week after the end of therapy to measure levels of total protein, albumin, glucose, total cholesterol, triglycerides and rapid-turnover proteins. Blood samples were taken from an antecubital vein after 30-min bed rest in the morning following an overnight fast. Plasma acyl-ghrelin and desacyl-ghrelin levels were measured by enzyme-linked immunosorbent assay (Mitsubishi Kagaku Iatron, Tokyo, Japan). Immunoradiometric assays were used to measure levels of serum GH (Ab Bead HGH Eiken; Eiken Chemical, Tokyo, Japan) and insulin-like growth factor (IGF)-1 (Somatomedin CII Bayer; Bayer Medical, Tokyo, Japan).

### Appetite assessment

Appetite in patients was quantified using the Edmonton Symptom Assessment Scale [6], which uses a 100-mm visual analog scale for appetite. Before and after 2-week administration of formula, appetite in patients was assessed before breakfast between 08:00 and 09:00.

### Statistical analysis

Data are expressed as mean ± standard deviation (SD). Comparison of ghrelin levels between the 2 groups was analyzed using the Mann-Whitney U test. Changes in parameters between the 2 groups were analyzed using the Wilcoxon signed-rank test. Values of p < 0.05 were taken to indicate statistical significance.

## Results

### Plasma ghrelin levels in patients with chronic pulmonary disease at study entry

Plasma acyl-ghrelin and desacyl-ghrelin levels were 11.0 ± 11.1 fmol/ml and 90.1 ± 52.4 fmol/ml, respectively, in the 19 outpatients with CRD (Table 1). Acyl-ghrelin levels trended to be lower and desacyl-ghrelin levels to be higher in patients than in controls (patients: 15.1 ± 12.9 fmol/ml; range, 4.0–42.5 fmol/ml and controls: 68.7 ± 62.0 fmol/ml; range, 20.5–197.5 fmol/ml, respectively), although no significant differences were identified. The sum of both forms of ghrelin was higher in patients (101.1 ± 58.8 fmol/ml) than in controls (83.7 ± 74.3 fmol/ml). The ratio of plasma acyl-ghrelin to desacyl-ghrelin was lower in patients (0.15 ± 0.16) than in controls (0.24 ± 0.10).

**Table 1:** Changes in parameters before and after 2-week once daily oral administration of octanoic acid formula to cachectic patients with chronic respiratory disease.

		Before	After	
body mass index	(kg/m^2^)	16.0 ± 2.00	16.3 ± 2.00	p < 0.05
appetite score		40 ± 22	64 ± 27	p < 0.05
acyl-ghrelin	(fmol/ml)	11.0 ± 11.1	14.8 ± 7.20	p < 0.05
desacyl-ghrelin	(fmol/ml)	90.1 ± 52.4	90.9 ± 52.5	NS
total protein	(g/dl)	6.9 ± 0.6	7.3 ± 0.7	p < 0.05
albumin	(g/dl)	3.8 ± 0.4	4.0 ± 0.4	p < 0.05
total cholesterol	(mg/dl)	181 ± 40	184 ± 210	NS
fasting glucose	(mg/dl)	94 ± 9	91 ± 90	NS
prealbumin	(mg/dl)	15.8 ± 4.20	17.9 ± 3.90	p < 0.05
transferrin	(mg/dl)	198 ± 41	231 ± 570	p < 0.05
retinol binding protein	(mg/dl)	1.9 ± 0.4	2.3 ± 0.5	p < 0.05
adrenalin	(pg/ml)	63 ± 40	60 ± 21	NS
noradrenalin	(pg/ml)	852 ± 320	724 ± 298	NS
dopamine	(pg/ml)	24 ± 10	18 ± 60	NS
GH	(ng/ml)	1.2 ± 1.0	1.3 ± 1.1	NS
IGF-1	(ng/ml)	87 ± 36	98 ± 39	p < 0.05

Age: range 62–78 y; sex:8 women, 11 men; oral 400 ml octanoic acid formula administered once daily after breakfast in addition to food intake of 1,800 kcal

### Ghrelin 24-h profiles with and without single administration of formula

**Figure 1:**
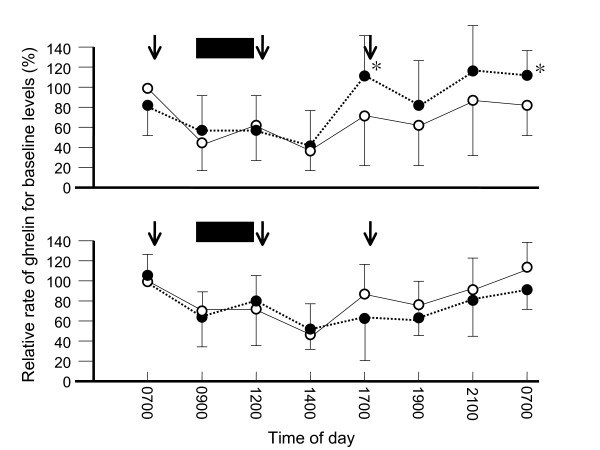
**24-h profiles of plasma acyl-ghrelin (upper) and desacyl-ghrelin (lower)**. Plasma ghrelin levels peaked in the early morning and decreased after meals. Plasma ghrelin levels are shown based on a value calculated before breakfast on the morning of day 1 as 100. Plasma desacyl-ghrelin levels with formula resembled those with no formula administration, while single administration of formula between breakfast and lunch induced higher acyl-ghrelin levels before dinner, remaining high until the next morning. Open circles, levels without administration of formula; closed circles, levels with administration of formula; closed squares administration of formula. Arrows show meal-taking for inpatients with chronic respiratory disease. Data are expressed as mean ± standard deviation. *, p < 0.05 for ghrelin level with vs. without administration of formula.

Plasma ghrelin levels peaked in the early morning and decreased after meals, supporting the findings of previous reports (Figure 1). Plasma desacyl-ghrelin levels with formula resembled those with no formula administration, while single administration of 400 ml of formula between breakfast and lunch induced higher acyl-ghrelin levels before dinner, remaining high until the next morning.

### Effect of 2-week administration of formula on plasma ghrelin, appetite, weight, nutrition status and hormone levels

Significant increases were seen in levels of plasma acyl-ghrelin, appetite score and body weight, but not desacyl-ghrelin. Levels of serum total protein, albumin and rapid turnover proteins increased after two-week administration of formula. No correlations were identified between the increases in acyl-ghrelin levels and weight or nutrition parameters. Two-week administration of formula did not alter fasting glucose, total cholesterol, triglyceride, catecholamines or GH levels, but induced an increase in serum IGF-1 levels.

## Discussion

This is the first paper showing a molecular analysis of plasma ghrelin in cachectic patients with CRD. Ghrelin profiles during the study showed that the total level of acyl-ghrelin and desacyl-ghrelin was high, but the ratio of plasma acyl-ghrelin to desacyl-ghrelin was low in cachectic CRD patients. High levels of ghrelin in cachectic patients have been suggested to maintain energy balance to prevent weight loss, consistent with previously studies reporting an inverse correlation between BMI and plasma ghrelin levels [7,8]. Acylation is necessary for ghrelin to induce appetite and desacyl-ghrelin is likely to inhibit appetite in mice [9], suggesting that the ratio of acylated ghrelin to desacyl-ghrelin may be important in determining the orexigenic effects of ghrelin.

The present study showed that administration of formula containing high levels of octanoic acids increased plasma acyl-ghrelin levels along with weight in patients with CRD. The study was designed for outpatients and exact food intake including formula during the 2-week period was not measured. Weight gain may have been due to the additional energy provided by the formula in addition to regular meals. In the present study, 2-week administration of the formula induced an increase in both weight and plasma acyl-ghrelin levels, suggesting that weight gain was associated with increases in acyl-ghrelin and the orexigenic effect was due to decreased plasma ghrelin levels when the patients displayed weight increases.

Additional induction of acyl-ghrelin induced a significant increase in IGF-1 levels. The concentration of circulating IGF-1 declines with age [10] and this hormone is involved in physiological changes of aging such as increased cardiovascular risk, reduced muscle mass and strength, reduced exercise tolerance and impaired quality of life [11]. IGF-1 stimulates osteoblast proliferation as well as osteoclast differentiation to inhibit osteopenia [12]. CRD with airflow obstruction has been shown to represent a causative risk for osteoporosis [13], so elevation of IGF-1 levels may be particularly useful for elderly individuals with CRD.

In conclusion, formula containing octanoic acids increased body weight and plasma acyl-ghrelin levels. This is the first trial showing a change in orexigenic hormone among patients receiving nourishment treatment. The present results seem likely to contribute to nutritional management in patients with cachectic diseases.

## Abbreviations

CRD: chronic respiratory disease; GH: growth hormone; IGF-1: insulin-like growth factor-1; BMI: body mass index.

## Competing interests

The authors declare that they have no competing interests.

## Authors' contributions

JA participated in study design, data analysis and manuscript preparation. NM participated in data collection and data analysis. MN participated in manuscript preparation and editing. All authors read and approved the final manuscript.
